# Multimodal analysis of TAAD pathogenesis: SHAP-enhanced interpretable models and single-cell sequencing analysis reveal immune microenvironment alterations

**DOI:** 10.3389/fimmu.2025.1630404

**Published:** 2026-01-14

**Authors:** Zhong Wang, Yixian Wang, Dianjun Tang, Qingwei Gang, Shikai Shen, Hongming Wei, Dongwen Zhao, Jian Zhang

**Affiliations:** 1 Department of Vascular and Thyroid Surgery, The First Hospital, China Medical University, Shenyang, Liaoning, China; 2 Department of Vascular Surgery, Wafangdian Central Hospital, Dalian, Liaoning, China; 3 Department of Vascular Surgery, The Second Hospital of Shandong University, Jinan, China

**Keywords:** immune microenvironment, inflammation, machine learning, SHAP, SIX4, Stanford type A aortic dissection

## Abstract

**Background:**

Stanford type A aortic dissection (TAAD) is a fatal cardiovascular emergency with high mortality within 48 hours. Elucidating molecular mechanisms and identifying reliable biomarkers are essential for improving diagnosis and guiding targeted interventions.

**Methods:**

We integrated four transcriptome datasets and two single-cell transcriptomic datasets using Harmony batch correction. Differentially expressed genes were identified with DESeq2. Three machine learning algorithms, LASSO, random forest, and SVM-RFE, were employed to identify hub genes, and SHAP analysis was used to quantify their individual contributions. A diagnostic system incorporating seven algorithms was constructed. Immune infiltration profiling, cell-cell communication analysis, and pseudotime trajectory analysis were performed. The proliferation and migration of vascular smooth muscle cells (VSMCs) were assessed using CCK-8 and wound healing assays.

**Results:**

Integration of bulk and single cell transcriptomic datasets identified three hub genes, SIX4, SCNN1B, and PCDH11X, through convergent machine learning approaches. SHAP analysis highlighted SIX4 as the predominant predictor within diagnostic models, which consistently achieved high accuracy (AUC > 0.9). Single cell profiling localized SIX4 expression to synthetic vascular smooth muscle cells, where it was linked to enhanced CXCL12–CXCR4 mediated immune interactions and remodeling of the inflammatory microenvironment. Functional assays confirmed that SIX4 overexpression promoted vascular smooth muscle cell proliferation and migration, corroborating its role in TAAD progression.

**Conclusion:**

This study uncovered SIX4, SCNN1B, and PCDH11X as critical regulators of TAAD. SIX4 was identified as a key modulator of smooth muscle cell plasticity and immune signaling dynamics. These findings deepen our understanding of TAAD pathogenesis and demonstrate the utility of SHAP-guided models in identifying and prioritizing mechanistic drivers in this complex vascular disease.

## 1 Introduction

Machine learning models are capable of identifying key genes and biomarkers closely associated with the onset and progression of diseases by integrating and analyzing high-throughput transcriptomic data (1). However, despite the significant predictive potential demonstrated by machine learning models, their “black box” nature often limits clinicians’ understanding and trust in the models’ decision-making processes (2). To address this, the SHapley Additive exPlanations (SHAP) interpretability method has emerged, which, based on the Shapley value. Stanford type A aortic dissection (TAAD), an acute tear in the ascending aortic intima with false lumen formation, is a lethal cardiovascular emergency. The mortality rate escalates rapidly, increasing by 1-2% per hour within the first 48 hours, and exceeding 50% within two days if surgical intervention is not undertaken (3). Despite urgent aortic replacement, 30-day mortality remains 10–30%, compounded by postoperative stroke, organ failure, and long-term aortic degeneration (4, 5). Current treatments rely on mechanical repair but fail to address underlying aortic vulnerability. Emerging evidence links TAAD pathogenesis to genetic mutations (6), hemodynamic stress (7), and matrix metalloproteinase-driven medial degeneration (8, 9). Deciphering these mechanisms is critical for developing biomarkers to guide early intervention and targeted therapies to stabilize the aortic wall, ultimately improving survival in this high-risk population (10, 11).

Machine learning technology is increasingly recognized as a pivotal instrument in contemporary medical research (1, 2, 12), facilitating the analysis of complex biomedical data and elucidating the molecular mechanisms underlying diseases (13). The integration of machine learning with the SHAP methodology facilitates the development of highly accurate predictive models for diseases, while simultaneously enhancing the comprehension of the impact of various risk factors on prediction outcomes. This approach thereby offers more transparent and credible support for clinical decision-making (14, 15). In this study, we aim to construct predictive models for TAAD by integrating transcriptomic data with machine learning algorithms, employing SHAP analysis to evaluate feature importance and model interpretability, identify TAAD-associated hub genes, and preliminarily investigate their molecular mechanisms using single-cell sequencing datasets.

## 2 Materials and methods

### 2.1 Dataset collection

Transcriptomic data were sourced from four publicly accessible transcriptome datasets available in the Gene Expression Omnibus (GEO) database (https://www.ncbi.nlm.nih.gov/) (16), comprising two RNA-seq datasets (GSE147026 and GSE153434) and two microarray datasets (GSE190635 and GSE52093). Additionally, single-cell RNA sequencing (scRNA-seq) data were retrieved from two independent GEO datasets (GSE213740 and GSE222318). All datasets were downloaded in their raw format for subsequent uniform preprocessing and analysis.

### 2.2 Batch effect correction and principal component analysis

The methodology has been refined to delineate that RNA-seq data, represented as raw counts, were processed using the rlog transformation provided by DESeq2. In contrast, normalized expression matrices derived from microarray datasets were utilized directly following the conversion of gene symbols and subsequent filtering. Upon the generation of these normalized expression matrices, all gene identifiers were standardized to official gene symbols, and a common gene set was established by determining their intersection. These matrices were then amalgamated into an initial integrated expression matrix. To address batch effects stemming from the disparate technological platforms (RNA-seq versus microarray) and various study sources, the ComBat function from the ‘sva’ R package was employed on this integrated matrix, with “platform” and “study GSE accession” designated as known batch variables. The adjusted matrix was subsequently utilized for all ensuing integrated analyses. The batch-corrected data were then subjected to principal component analysis (PCA) using the prcomp R package (v3.6.3) to evaluate the effectiveness of batch effect removal. PCA was conducted on the 2,000 most highly variable genes (selected by mean-variance relationship) using singular value decomposition (SVD).

### 2.3 Differential expression and function enrichment analysis

Following successful integration and batch effect correction, we employed the linear model-based limma package for differential expression analysis. This method is particularly suitable for processing continuous expression data that has been transformed to approximate a normal distribution. Significantly differentially expressed genes were identified using a screening threshold of |log2 fold change| > 1 and FDR-adjusted p-value < 0.05. To functionally characterize DEGs, we performed enrichment analyses through the clusterProfiler toolkit (v4.0), encompassing Gene Ontology (GO) and Kyoto Encyclopedia of Genes/Genomes (KEGG) pathways. GO evaluation comprehensively examined three ontological categories - Biological Processes, Molecular Functions, and Cellular Components - employing hypergeometric testing with Benjamini-Hochberg FDR adjustment (FDR<0.05) to determine significance (17). Concurrent KEGG profiling detected altered metabolic/signaling pathways, applying equivalent significance criteria for pathway validation (18).

### 2.4 Feature selection using machine learning approaches

Three machine learning methods were selected for their complementary strengths: LASSO for feature shrinkage and multicollinearity control, Random Forest for robustness to noise and non-linear interactions, and SVM-RFE for recursive feature elimination with high-dimensional data. All models were developed and evaluated under a stratified nested cross-validation framework to ensure robust performance estimation and mitigate overfitting.

#### 2.4.1 LASSO regression analysis

Lasso regression analysis constitutes a shrinkage and variable selection technique employed in the context of linear regression models (19, 20). This analysis employs a machine learning algorithm in conjunction with bias estimation to effectively address multicollinearity and facilitate the selection of relevant variables. Following the shrinkage process, variables with regression coefficients of zero are excluded from the model, whereas those with non-zero regression coefficients demonstrate a significant association with the response variable. Explanatory variables may be quantitative, categorical, or a combination of these types. L1-penalized LASSO regression was implemented through the glmnet package (v4.1) for concurrent feature selection and overfitting control. The optimal regularization parameter (λ) was determined via 10-fold cross-validation, selecting the value that minimized the binomial deviance (21, 22). Across 100 λ iterations along the regularization path, genes demonstrating non-zero coefficients at optimal λ were selected as predictive biomarkers.

#### 2.4.2 Random forest modeling

Random Forest is regarded as one of the most influential algorithms in the global field of machine learning. In contrast to traditional linear regression models, this algorithm utilizes an ensemble of multiple regression trees to reduce the risk of overfitting. It offers several distinct advantages, including improved model interpretability, enhanced capability for processing multiple features, easily extendable classification functionality, and scale-free characteristics. The algorithm is capable of executing data mining, classification, and regression tasks, thus facilitating the identification of more nuanced correlations among variables (23). A random forest classifier was constructed using the randomForest package (v4.7) with 500 decision trees (https://cran.r-project.org/package=randomForest). The model was constructed with 500 trees (ntree=500), and variable importance was assessed using the mean decrease in Gini impurity. Model stability was evaluated by plotting learning curves over incremental training subsets.

#### 2.4.3 SVM-RFE feature selection

Support Vector Machine-Recursive Feature Elimination (SVM-RFE) was employed in conjunction with 10-fold cross-validation to iteratively optimize the feature subset. The algorithm systematically assessed model efficacy across incremental feature subsets, identifying the configuration achieving superior predictive performance through minimized cross-validation error and maximized classification accuracy. Feature selection procedures were conducted utilizing a five-repeat 80/20 train-test split methodology. The feature elimination process was coupled with 10-fold cross-validation to identify the feature subset that yielded the minimal cross-validation error. The stability of the selected features was evaluated through bootstrap resampling with 100 iterations. Genes that were consistently selected in more than 80% of these iterations were retained for further analysis.

### 2.5 SHAP analysis

To elucidate the biological relevance of selected features, SHAP analysis was conducted using the shapviz package (v0.9). This algorithm calculates the SHAP value to show the size and direction of features’ impact on models, providing both local and global interpretability analyses for the specific classification. Local interpretability analysis reveals how each feature contributes to the prediction for a particular sample, where the output is elevated from the base value to the final value. Global feature importance was quantified by averaging absolute SHAP values across all samples, and top-ranked genes were visualized in descending order of their mean SHAP scores.

### 2.6 Consensus gene identification

Genes consistently identified by all three machine learning methods were considered high-confidence candidates. The intersection was determined using the VennDiagram package (v1.7). Chromosomal locations of candidate genes were mapped using GRCh38/hg38 assembly coordinates from the UCSC Genome Browser. We quantified gene contributions using SHAP values implemented in the shapviz package (v0.9). Mean absolute SHAP values were calculated across all samples.

### 2.7 Consensus gene-based diagnostic model construction and validation

Predictive models were conducted using seven machine learning algorithms, including partial least squares (PLS), RF, Decision Tree with Shrinkage (DTS), SVM, Logistic Regression, K-Nearest Neighbors (KNN) and eXtreme Gradient Boosting (XGBoost), to evaluate the diagnostic potential of three consensus genes identified through prior feature selection. All models were uniformly trained on standardized gene expression profiles (mean-centered and unit-variance scaled) under a stratified nested cross-validation framework, comprising 10-fold data partitioning with 5 repetitions and hyperparameter optimization via grid search. The primary metric for evaluating model accuracy was the Area Under the Curve (AUC), which was derived from Receiver Operating Characteristic (ROC) analysis performed with the pROC package.

### 2.8 Immune microenvironment profiling

Immune cell composition and gene-cell interactions were analyzed using CIBERSORTx (v1.0) with the LM22 leukocyte signature matrix (24). Raw transcriptomic data underwent ComBat batch correction, TMM normalization, and filtration (genes retained with ≥1 count per million in >50% samples) prior to deconvolution. Spearman’s rank correlation analysis with Benjamini-Hochberg FDR adjustment quantified pairwise associations among 22 immune cell subsets, where correlations with |ρ| ≥0.5 and FDR <0.01 were considered significant.

### 2.9 Functional pathway enrichment

Gene set enrichment analysis (GSEA) was conducted on the KEGG pathways (version 2023.1) utilizing the clusterProfiler package (version 4.0). Samples were stratified into SIX4 high- and low-expression groups based on median expression cutoff. Enrichment significance was assessed through 1,000 phenotype permutations, retaining pathways meeting FDR <0.25 and normalized enrichment score (NES) |>1.5|. A relaxed FDR threshold (<0.25) was used for GSEA to capture broader biological trends, as recommended for pathway enrichment analysis in exploratory studies. Leading-edge analysis identified core gene subsets driving pathway enrichment. Results were visualized via enrichment plots highlighting divergent biological processes between expression groups.

### 2.10 Dimensionality reduction and clustering

Single-cell RNA sequencing data preprocessing included quality control (mitochondrial gene percentage <20%, detected genes >500), normalization via SCTransform, and selection of 3,000 highly variable genes. Raw expression matrices from different platforms were normalized using the Harmony algorithm (v1.0) to remove technical batch effects while preserving biological variability. Dimensionality reduction was performed using both t-distributed stochastic neighbor embedding (t-SNE, perplexity=30, theta=0.5) and uniform manifold approximation (UMAP, n_neighbors=15, min_dist=0.1) algorithms. Cell clustering was achieved through graph-based Louvain clustering (resolution=0.8) on the shared nearest neighbor graph (k=20) constructed in PCA space.

### 2.11 Intercellular communication analysis

Cell-cell interaction networks were reconstructed using CellChat (v1.6.0) with its default ligand-receptor interaction database. The communication probability between cell types was systematically computed through a multi-step approach involving trimean aggregation of ligand and receptor expression levels, incorporation of spatial proximity information with a weighting factor (α = 0.75), and statistical validation via permutation testing (n = 1,000 iterations), with interactions considered statistically significant at a false discovery rate (FDR) of < 0.01. This comprehensive framework ensured robust identification of biologically relevant intercellular signaling events.

### 2.12 Pseudotime trajectory inference

Pseudotime trajectory inference was performed using Monocle3 (v1.3.1), wherein cell differentiation dynamics were reconstructed through a computational framework that included UMAP-based trajectory graph construction to embed cellular relationships in reduced dimensional space, principal graph learning with a topological constraint (max_components = 3) to identify major branching points of phenotypic transitions, and pseudotemporal ordering via reversed graph embedding to align cells along inferred differentiation paths.

### 2.13 Western blot analysis

Protein expression levels of SIX4 were evaluated by western blot analysis. Total proteins were extracted from tissues or cultured cells using RIPA lysis buffer supplemented with protease inhibitors. After quantification with a BCA assay, equal amounts of protein were separated by SDS-PAGE and transferred onto PVDF membranes. The membranes were blocked with 5% non-fat milk in TBST for 1 hour at room temperature and then incubated overnight at 4 °C with a primary antibody specifically targeting SIX4 (ab176713, Abcam, Cambridge, UK; dilution 1:1000). After washing, the membranes were probed with an HRP-conjugated secondary antibody for 1 hour at room temperature. Protein bands were visualized using an ECL chemiluminescent substrate (BL520A, Biosharp, China) and imaged with a chemiluminescence imaging system (Tano 5200Multi). Band intensities were quantified using ImageJ software, normalized to GAPDH as a loading control.

### 2.14 Quantitative real-time PCR analysis

Total RNA was isolated using the SevenFast Total RNA Extraction Kit (SM132, China). cDNA synthesis and subsequent qPCR amplification were performed with the Takara ExTaq PCR kit (639505, Japan) according to the manufacturer’s protocol. Amplification data were processed using Applied Biosystems’ QuantStudio™ Design & Analysis Software. The primer sequences for SIX4 detection were:

Forward: 5’-CGAGCTCTACAGCATCCTCG-3’;

Reverse: 5’-CGGTACTTGTCTACGGCTCC-3’.

### 2.15 Cell transfection

VSMCs purchased from ScienCell (CA, USA), were cultured in DMEM medium supplemented with 10% fetal bovine serum until reaching approximately 70% confluency. Transfection of the SIX4 overexpression plasmid into VSMCs was conducted using Lipo3000 liposomes (Invitrogen, Cat. No. L3000015, USA). VSMCs purchased from ScienCell (CA, USA) were cultured in DMEM supplemented with 10% fetal bovine serum (FBS) and 1% penicillin/streptomycin at 37 °C in a 5% CO_2_ humidified atmosphere. The cells were passaged when reaching approximately 80–90% confluency using 0.25% trypsin-EDTA and were used for experiments within passages 3–6. For transfection, VSMCs were seeded into appropriate culture vessels and allowed to grow until approximately 70% confluency. The SIX4 overexpression plasmid was transfected into VSMCs using Lipofectamine™ 3000 transfection reagent (Invitrogen, Cat. No. L3000015, USA) according to the manufacturer’s instructions. Briefly, plasmid DNA and Lipofectamine 3000 were diluted separately in Opti-MEM^®^ reduced serum medium, mixed gently, incubated for 15 minutes at room temperature to form complexes, and then added dropwise to the cell cultures. After 6 hours of transfection, the medium was replaced with fresh complete medium to reduce cytotoxicity. Cells were harvested 48 hours post-transfection for subsequent functional validation experiments.

### 2.16 Wound healing assay

Cell migration capacity was evaluated using a wound healing assay. After VSMCs reached complete confluence in 6-well plates, a standardized scratch was created using a pipette tip. Following medium replacement, wound closure was monitored by capturing images at 0 h and 24 h post-scratch under 200× magnification (25). Migration rates were quantified by measuring the reduction in scratch area between time points using ImageJ software.

### 2.17 Cell counting kit-8 assay

After 12–24 hours of transfection, cells in good growth condition were collected and routinely digested, followed by cell counting. The cells were seeded into 96-well plates at a density of 2,000 cells per well, with 100 µL of culture medium added to each well. Six replicate wells were set for each group. At 0 h, 24 h, 48 h, 72 h, and 96 h post-seeding, 10 µL of CCK-8 solution was added to each well. The absorbance at 450 nm was measured using a microplate reader. A growth curve was plotted with time on the x-axis and OD values on the y-axis.

### 2.18 Statistical analysis and visualization

All statistical analyses were performed using R (version 4.2.2) and Python (version 3.9). For differential expression analysis, we applied negative binomial generalized linear models (implemented in DESeq2) and non-parametric Wilcoxon rank-sum tests. Multiple testing correction was conducted using the Benjamini-Hochberg method to control the false discovery rate (FDR), with a significance threshold set at P<0.05.

## 3 Results

### 3.1 Batch effect correction and differential gene expression analysis in TAAD datasets

**Figure 1:**
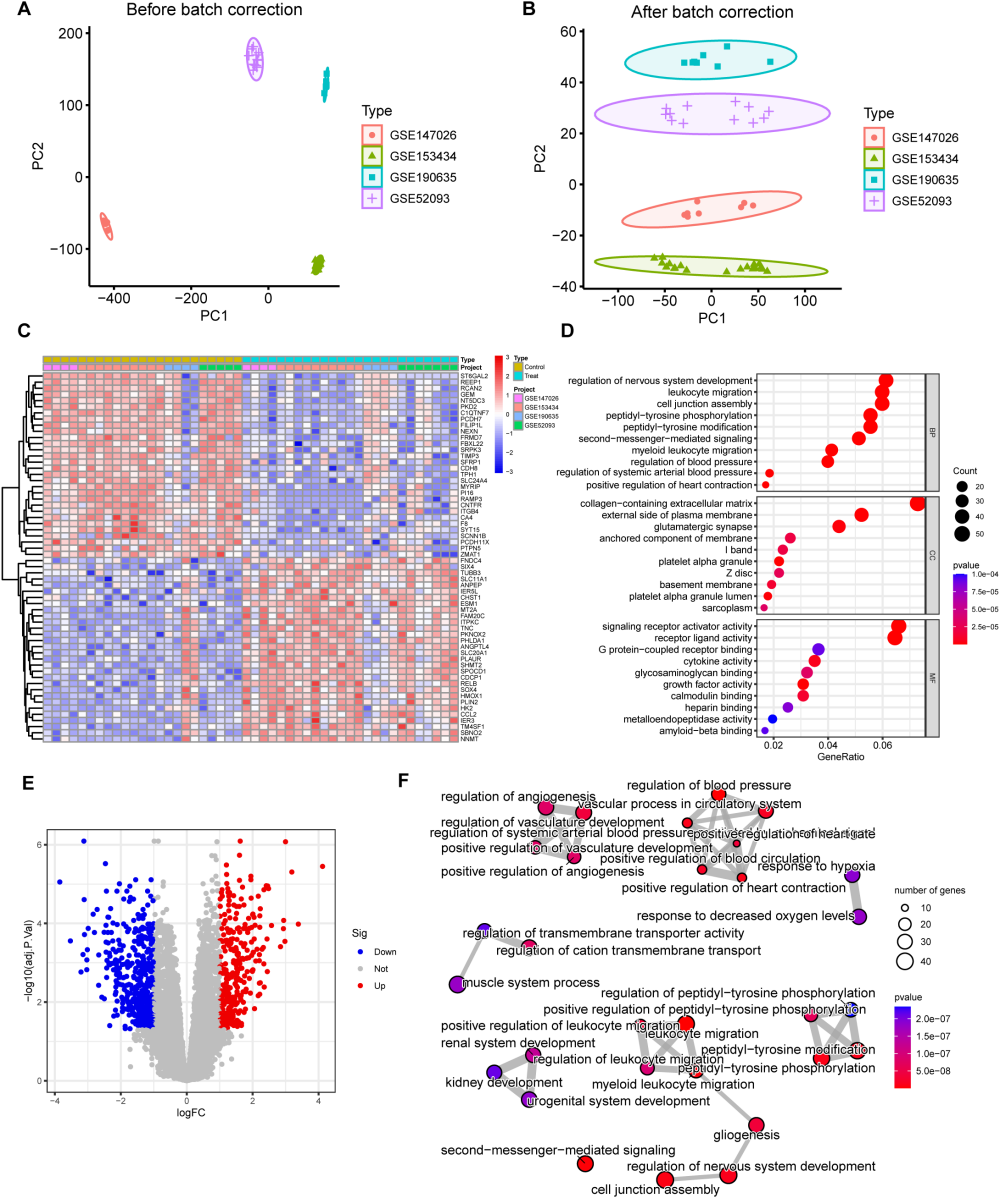
Batch effect correction and functional analysis of TAAD transcriptomic datasets. **(A)** PCA of four GEO datasets (GSE147026, GSE153434, GSE190635, GSE52093) before batch correction. **(B)** PCA plot after Harmony batch effect correction. After correcting batch effects with harmony methods, PC1 was significantly reduced, and within-group dispersion decreased, indicating that batch-related technical variation was largely eliminated. **(C)** Heatmap of the top 60 differentially expressed genes (DEGs) (top 30 upregulated and top 30 downregulated) between TAAD and control groups. Rows represent Z-score-normalized gene expression (blue-to-red gradient). **(D)** GO enrichment of DEGs. **(E)** Volcano plot of DEGs (FDR < 0.05, |log2FC| > 1). **(F)** KEGG pathway enrichment results for DEGs.

Before correction, the four datasets (GSE147026, GSE153434, GSE190635, GSE52093) exhibited clear separation by source in principal component space, indicating strong batch effects (**Figure 1A**). The results demonstrate significant improvement after ComBat correction: PC1 variance explained decreased from 54.94% to 14.51% (40.43 percentage point reduction). PC2 variance explained decreased from 18.67% to 8.32% (10.35 percentage point reduction). Cumulative variance explained by PC1 and PC2 decreased from 73.62% to 22.82%. After batch effect correction using harmony methods, PC1 was significantly reduced, and within-group dispersion markedly decreased, suggesting that batch-related technical variation had been largely eliminated (**Figure 1B**). **Figure 1C** illustrates the 60 genes exhibiting the most significant differential expression between the TAAD and control groups, comprising the 30 most upregulated and 30 most downregulated genes. In the TAAD group, there was a pronounced upregulation of genes such as FNDC4, SIX4, and TUBB3, whereas ST6GAL2, REEP1, and RCAN2 were among the most significantly downregulated. Samples were clustered by group, and genes were hierarchically clustered by expression patterns. The color gradient represents Z-score-normalized expression levels. **Figure 1E** presents a volcano plot of differentially expressed genes, analyzed using DESeq2 with significance thresholds set at FDR < 0.05 and |log2FC| > 1. Gray dots represent non-significant genes, red dots indicate genes significantly upregulated in the TAAD group, and blue dots denote genes significantly downregulated in the TAAD group. GO enrichment analysis revealed that DEGs were significantly enriched in regulation of nervous system development, leukocyte migration, collagen-containing extracellular matrix, external side of plasma membrane, signaling receptor activator activity, and receptor ligand activity (**Figure 1D**). KEGG pathway analysis (**Figure 1F**) showed that DEGs were significantly enriched in multiple signaling pathways. The most prominent pathways identified include the regulation of blood pressure, vascular processes within the circulatory system, regulation of nervous system development, and the positive regulation of peptidyl-tyrosine phosphorylation. Collectively, these enriched pathways underscore significant hemodynamic alterations, particularly the aberrant activation mediated by phosphorylation, which may directly contribute to structural changes in the vasculature of the circulatory system. This further implicates their role in the pathogenesis of TAAD.

### 3.2 Machine learning-based identification of TAAD signature genes

**Figure 2:**
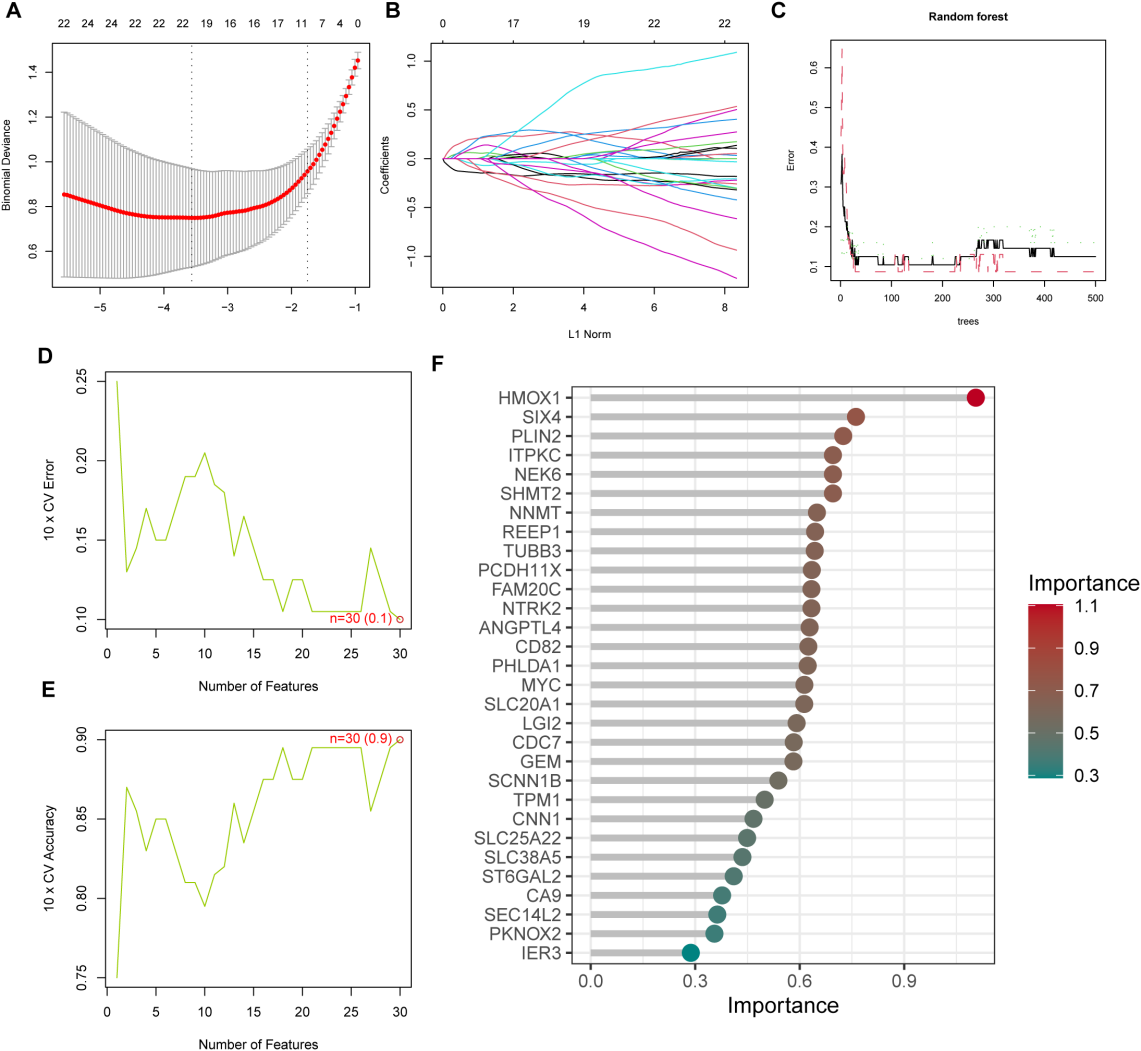
Machine learning identification of TAAD signature genes. **(A)** LASSO coefficient profiles with varying λ values (10-fold cross-validation). **(B)** L1 regularization path analysis of LASSO regression. **(C)** Random forest model learning curve. **(D, E)** SVM-RFE feature selection performance. **(F)** SHAP analysis of selected feature genes.

Through LASSO regression analysis, we systematically screened the most relevant feature genes for TAAD. The regularization path analysis showed that as the regularization parameter λ increased, the number of genes retained by the model gradually decreased. The optimal λ value was determined through 10-fold cross-validation, achieving effective feature compression while maintaining low binomial deviance (**Figure 2A**). Ultimately, LASSO regression identified 21 core genes with non-zero coefficients (**Figure 2B**). The learning curve of the random forest model (**Figure 2C**) demonstrated that as the training sample size increased, the model’s accuracy progressively improved and stabilized, with no evidence of overfitting. Using the SVM-RFE method, we evaluated model performance with different numbers of features through 10-fold cross-validation. The results showed that the cross-validation error reached the minimum when the number of features was 30 (**Figure 2D**), indicating that further increasing the number of features might lead to overfitting. The classification accuracy was highest with 30 features (**Figure 2E**), outperforming other feature scales. Through the SHAP interpretability method, we conducted a global importance assessment of the 30 key feature genes selected by SVM-RFE and visualized them using a bar chart (**Figure 2F**). Genes such as HMOX1, SIX4, and PLIN2 ranked highest in weight scores.

### 3.3 Identification of core TAAD-associated genes through machine learning integration

**Figure 3:**
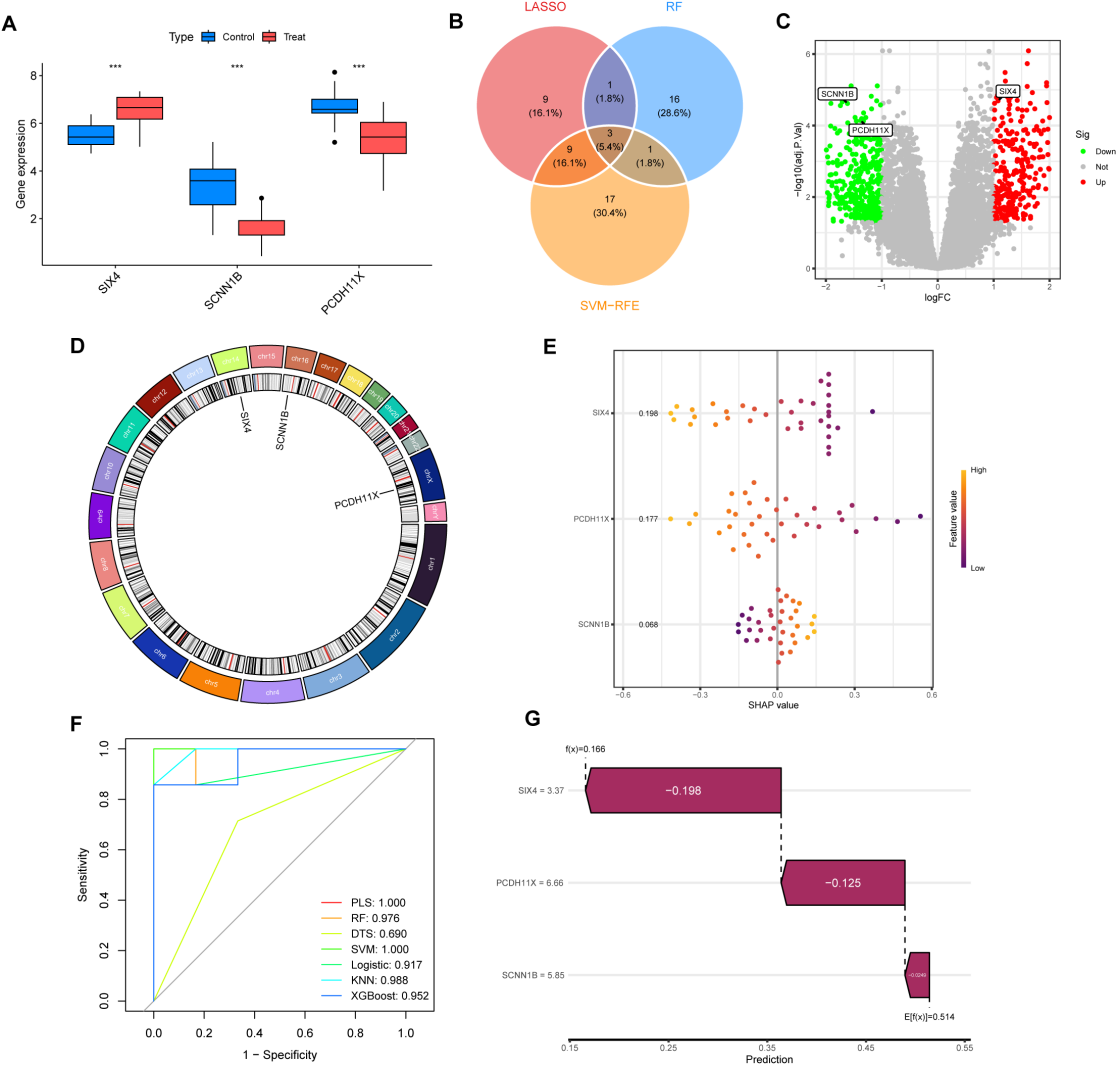
Identification and validation of core TAAD-associated genes through machine learning integration. **(A)** Venn diagram of overlapping genes identified by LASSO, random forest (RF), and SVM-RFE analyses. **(B)** Violin plots of SIX4, SCNN1B, and PCDH11X between TAAD and control groups. **(C)** Volcano plot highlighting the three consensus genes. **(D)** Genomic locations of SIX4, SCNN1B and PCDH11X. **(E, G)** SHAP analysis ranking gene contributions to model predictions. **(F)** ROC curves of models incorporating the three genes. SVM-RFE: Support Vector Machine-Recursive Feature Elimination. PLS: partial least squares. RF: Random Forest. DTS: Decision Tree with Shrinkage. SVM: Support Vector Machine. Logistic: Logistic Regression. KNN:K-Nearest Neighbors. XGBoost:eXtreme Gradient Boosting.

By integrating feature genes identified through three machine learning approaches (LASSO, random forest, and SVM-RFE), we identified three core overlapping genes: SIX4, SCNN1B, and PCDH11X (**Figure 3A**). Genes selected by each method are listed in **Supplementary Table S1**. Bootstrap resampling (n=100) confirmed the stability of SIX4, SCNN1B, and PCDH11X, with selection frequencies >85%. Violin plots (**Figure 3B**) demonstrated that compared to normal controls, SIX4 expression was significantly upregulated (P < 0.001) in the TAAD group, while SCNN1B and PCDH11X were significantly downregulated (P < 0.001), consistent with their positions in the volcano plot (**Figure 3C**). The genomic locations of SIX4 (Chr14), SCNN1B (Chr16), and PCDH11X (ChrX) suggested they might be regulated by distinct mechanisms (**Figure 3D**). The SHAP value quantifies the magnitude and direction (positive or negative) of a feature’s impact on the model’s output for a single prediction. The mean absolute SHAP value across all samples represents a feature’s global importance. SHAP analysis revealed SIX4 made the greatest contribution to model predictions (mean SHAP value = 0.198), underscoring its role as the predominant predictor, followed by PCDH11X and SCNN1B (**Figures 3E, G**). ROC analysis indicated that machine learning models integrating these three genes exhibited exceptional performance in the classification of TAAD (**Figure 3F**). All models achieved AUC greater than 0.69, demonstrating robust performance. Notably, the PLS and SVM models both attained an AUC of 1.000, indicating perfect discrimination. The KNN model followed closely with an AUC of 0.988, while the RF model achieved an AUC of 0.976. The XGBoost model demonstrated strong performance with an AUC of 0.952, and the Logistic regression model also performed well with an AUC of 0.917. In contrast, the Decision Tree (DTS) model exhibited moderate discriminatory ability with an AUC of 0.690. Although all models were evaluated using stratified cross-validation to ensure robust performance estimation, the possibility of overfitting cannot be fully ruled out, particularly given the sample size constraints.

### 3.4 Immune cell correlations and gene expression patterns in TAAD microenvironment

**Figure 4:**
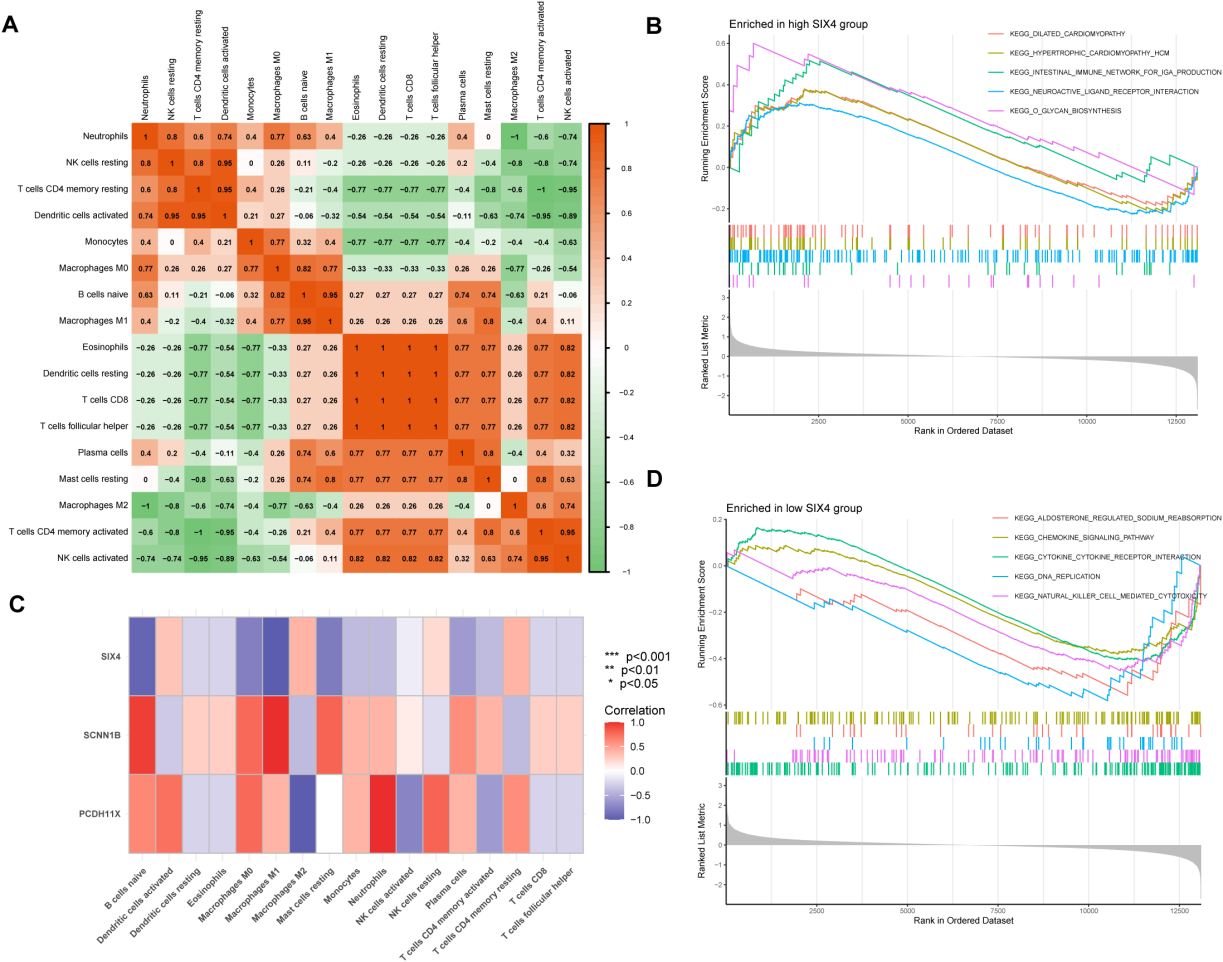
Immune microenvironment characterization and functional enrichment analysis in TAAD. **(A)**CIBERSORT analysis of immune cell populations in TAAD tissues. **(B)** GSEA showing top enriched KEGG pathways in SIX4 high-expression group. **(C)** Expression correlations of SIX4, SCNN1B and PCDH11X with immune cell subsets. **(D)** GSEA showing top enriched KEGG pathways in SIX4 low-expression group.

Analysis of immune cell composition in TAAD tissues using the CIBERSORT algorithm revealed significant correlations between immune cell populations, including a strong positive correlation between neutrophils and resting NK cells (r = 0.8) as well as between neutrophils and resting CD4 memory T cells (r = 0.95), indicating the presence of coordinated inflammatory responses within the TAAD microenvironment (**Figure 4A**). A detailed analysis of the expression patterns of the genes SIX4, SCNN1B, and PCDH11X across various immune cell subsets revealed distinct expression profiles (**Figure 4C**). Specifically, SIX4 was negatively correlated with both M1 macrophages and naive B cells, whereas SCNN1B demonstrated positive correlations with these cell types. In contrast, PCDH11X was positively correlated with neutrophils and negatively associated with M2 macrophages. Furthermore, GSEA comparing KEGG pathways between groups with high and low SIX4 expression levels identified significant differences in pathway activation (**Figures 4B, D**). The SIX4 high-expression group showed marked enrichment for pathways related to cardiac pathophysiology and immune regulation, along with O-glycan biosynthesis. In contrast, the SIX4 low-expression group was predominantly associated with pathways involving sodium reabsorption regulation, immune signaling, cellular proliferation, and immune effector functions.

### 3.5 Single-cell transcriptomics reveals SIX4^+^ SMCs as key mediators in TAAD

**Figure 5:**
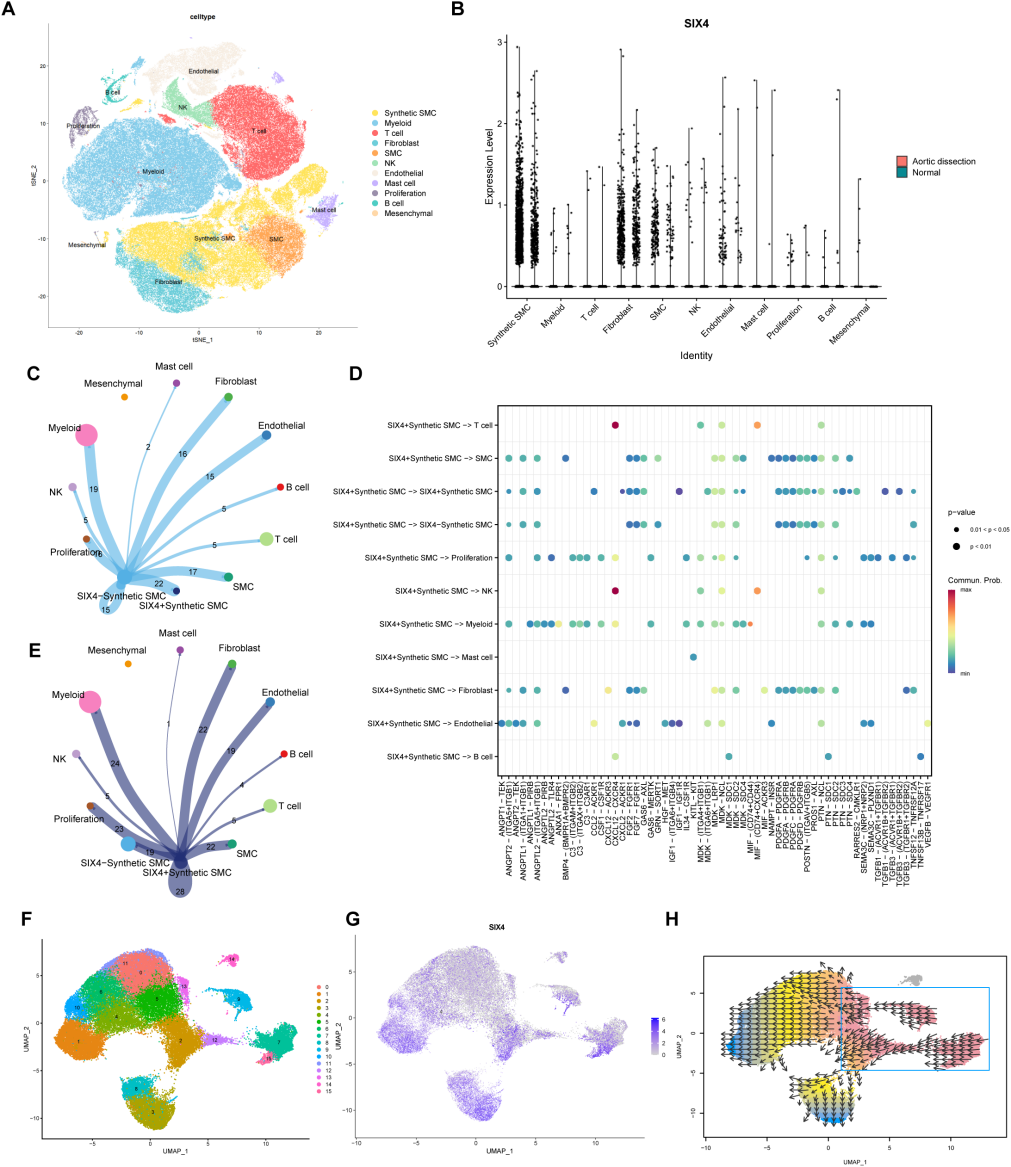
Single-cell transcriptomic profiling identifies SIX4+ synthetic SMCs as key players in aortic dissection pathogenesis. **(A)** t-SNE visualization of 11 major cell clusters identified in TAAD tissues. **(B)** Violin plots showing cell type-specific expression of SIX4. **(C)** Cellular interaction network of SIX4- synthetic SMCs with other cell types. **(D)** Ligand-receptor pair analysis between SIX4+ synthetic SMCs and interacting cells. The color of the bubbles represents the computed communication probability, indicating the strength of the signaling interaction. **(E)** Cellular interaction network of SIX4+ synthetic SMCs with other cell types. **(F)** UMAP subclustering of synthetic SMCs into 15 distinct subsets. **(G)** UMAP projection of SIX4 expression across synthetic SMC subclusters. **(H)** Pseudotime trajectory analysis of synthetic SMCs.

The t-SNE dimensionality reduction and clustering of single-cell RNA sequencing data identified 11 major cell populations (**Figure 5A**), including synthetic smooth muscle cells (SMCs), myeloid cells, T cells, fibroblasts, SMCs, natural killer (NK) cells, endothelial cells, mast cells, proliferating cells, B cells, and mesenchymal cells. Synthetic SMCs were defined by high expression of COL1A1 and low expression of contractile markers such as MYH11, consistent with a phenotypic switching phenotype. Subsequent analysis revealed cell type-specific expression of SIX4, showing highest expression in synthetic SMCs (**Figure 5B**) but lower levels in quiescent SMCs, suggesting its potential involvement in SMC phenotypic switching. Synthetic SMCs were further stratified into SIX4-negative and SIX4^+^ subsets based on SIX4 expression levels. The comparative analysis revealed that SIX4^+^ synthetic SMCs exhibited significantly stronger interaction intensity and a greater number of interaction pathways with various cell types, including SMCs (22 versus 17), endothelial cells (19 versus 15), and fibroblasts (22 versus 16), in comparison to SIX4- subsets. (**Figures 5C, E**). Ligand-receptor pair analysis identified particularly strong CXCL12-CXCR4-mediated interactions between SIX4^+^ SMCs and T/NK cells (**Figure 5D**). UMAP subclustering resolved synthetic SMCs into 15 distinct subpopulations (**Figure 5F**), with SIX4 expression patterns visualized across these subsets (**Figure 5G**). The pseudotime trajectory suggests a progression of synthetic SMCs towards a terminal state characterized by high SIX4 expression, consistent with a pathologically committed, pro-inflammatory, and synthetic phenotype (**Figure 5H**).

### 3.6 SIX4 is highly expressed in TAAD tissues and promotes the proliferation and migration of VSMCs

**Figure 6:**
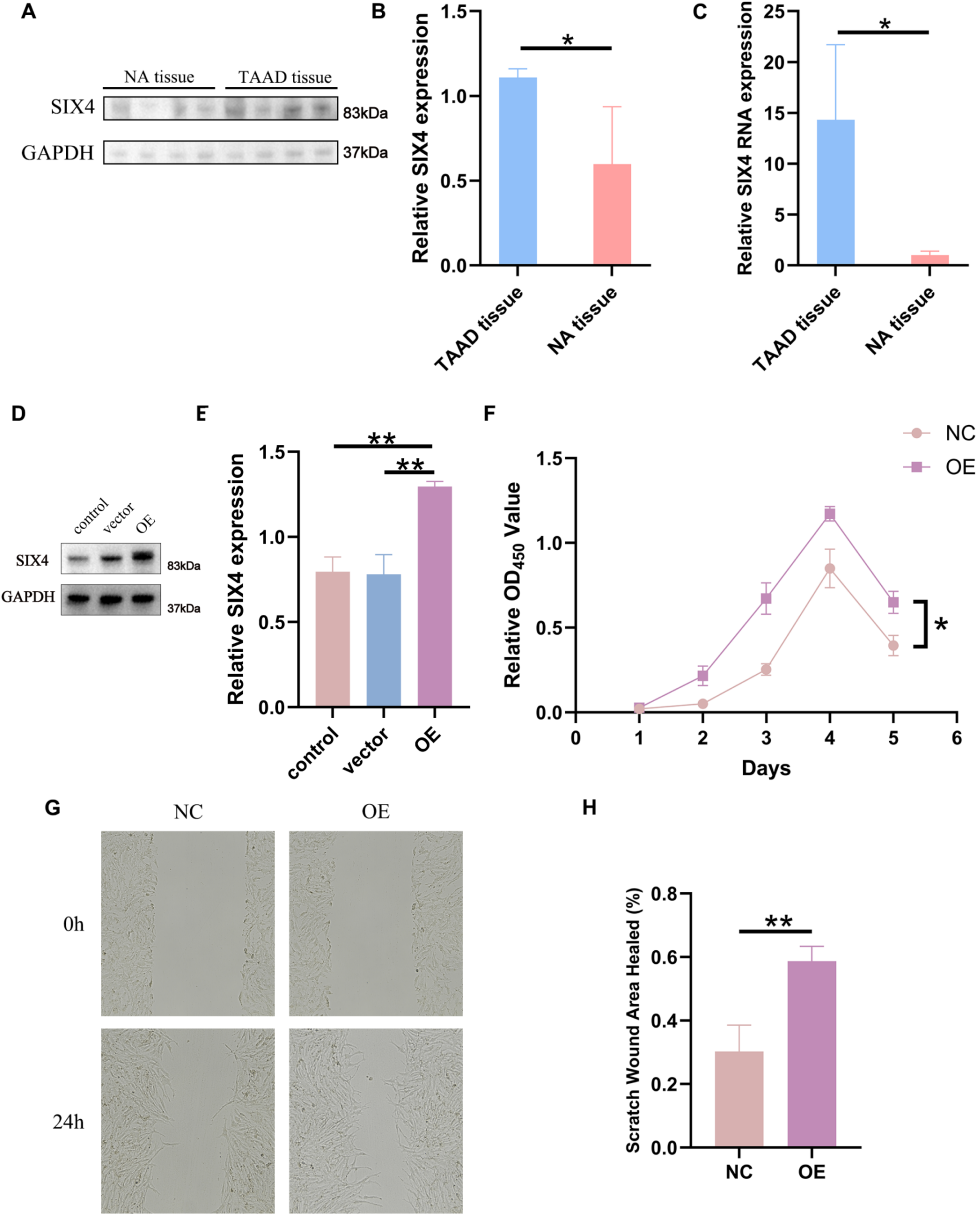
SIX4 is highly expressed in TAAD tissues and promotes the proliferation and migration of VSMCs. **(A)** WB gel images of SIX4 in four cases of TAAD tissues and four cases of normal aortic tissues. **(B)** Statistical graph of SIX4 expression levels relative to GAPDH. **(C)** PCR statistical results of SIX4 mRNA levels. **(D)** WB validation of SIX4 overexpression in VSMCs cell lines. **(E)** Statistical graph of SIX4 expression levels relative to GAPDH. **(F)** CCK8 results of the SIX4 overexpression group and the control group. **(G, H)** Wound healing assay results and statistical analysis of the SIX4 overexpression group and the control group. TAAD tissue: aortic dissection tissue; NA tissue: normal aortic tissue; control: blank group; vector: blank vector group; OE: SIX4 expression group; NC: control group. * p<0.05, ** p<0.01.

To validate the expression level of SIX4 in TAAD tissues, we collected four clinical samples of TAAD tissues from surgical patients and four normal aortic tissues obtained from liver transplant donors. Total RNA and protein were extracted from these tissues using TRIzol reagent and RIPA lysis buffer, respectively. The mRNA expression of SIX4 was detected by quantitative real-time PCR (qPCR), while protein expression was examined by Western blotting. The results demonstrated that SIX4 was significantly upregulated in TAAD tissues at both the transcriptional level (P < 0.05) and protein level (P < 0.05) compared with normal aortic tissues (**Figures 6A–C**). To further investigate the functional role of SIX4, human vascular smooth muscle cells (VSMCs) were transfected with a SIX4 overexpression plasmid. Western blot analysis confirmed that SIX4 protein expression was significantly increased in the transfection group compared with the control group (P < 0.01) (**Figures 6D, E**). CCK-8 assays indicated that the proliferation ability of VSMCs overexpressing SIX4 was significantly enhanced relative to the control group (P < 0.05) (**Figure 6F**). In addition, scratch wound healing assays showed that the migration area of VSMCs in the SIX4 overexpression group was significantly larger than that in the control group (P < 0.01) (**Figures 6G, H**), suggesting that SIX4 promotes VSMC migration.

## 4 Discussion

Stanford type A aortic dissection (TAAD) remains a catastrophic cardiovascular emergency characterized by abrupt intimal rupture, rapid hemodynamic deterioration, and high early mortality (26, 27). Despite advances in surgical management, the molecular underpinnings of TAAD pathogenesis remain incompletely understood, hindering early detection and personalized interventions. Recent studies have emphasized the critical role of VSMC phenotypic switching, immune infiltration, and extracellular matrix remodeling in aortic wall degeneration (28). However, most investigations have relied on single-layer omics or conventional statistical approaches, often lacking mechanistic resolution or predictive interpretability. In our study, we integrate bulk transcriptomics, single-cell RNA sequencing, and SHAP-guided machine learning to identify novel regulatory drivers and establish an interpretable diagnostic framework for TAAD. Moreover, the multimodal framework established in this study, which integrates SHAP-based interpretable machine learning with single-cell transcriptomics, extends beyond the analysis of immune alterations. It has the potential to illuminate additional aspects of the pathogenesis of TAAD, including genetic predispositions, hemodynamic stresses, and metabolic disturbances.

Our integrated machine learning pipeline, encompassing LASSO, random forest, and SVM-RFE algorithms, converged on three hub genes—SIX4, SCNN1B, and PCDH11X. Among these, SIX4 emerged as the top-ranked contributor across all SHAP analyses, suggesting a dominant role in TAAD pathophysiology. SIX4, a sine oculis homeobox transcription factor, has previously been implicated in organogenesis and tumor progression, particularly through AKT/HIF1α-mediated angiogenic signaling (29–31). However, its role in vascular biology and aortic disease had not been elucidated. Here, we demonstrate for the first time that SIX4 is specifically upregulated in synthetic VSMCs within the dissected aortic wall, and is functionally associated with enhanced proliferation and migration capacity—hallmarks of pathological phenotypic switching. The detection of SIX4 in plasma or its assessment via immunohistochemistry in aortic tissue holds promise as a potential biomarker for TAAD diagnosis, meriting further investigation into its clinical utility.

As a core subunit of the epithelial sodium channel (ENaC), the aberrant expression of SCNN1B in vascular smooth muscle may contribute to TAAD pathogenesis through sodium ion homeostasis dysregulation (32). ENaC modulates intracellular Na^+^ concentration, thereby indirectly regulating calcium signaling via the Na^+^/Ca^2+^ exchanger (NCX), which subsequently influences vascular tone and SMC contractile function (33). Prior research has shown that hypomethylation at the CpG1/CpG2 sites within the SCNN1B promoter region is associated with essential hypertension. Furthermore, the methylation status of these sites is influenced by antihypertensive treatment (34, 35). This indicates that the dysregulation of SCNN1B might contribute to the pathogenesis of hypertension by disrupting sodium ion homeostasis (36, 37). Notably, the observed SCNN1B downregulation in TAAD specimens potentially reflects a synergistic pathogenic mechanism between vascular smooth muscle ion channel dysfunction and hemodynamic disturbances (38, 39).

We also identified the PCDH11X expression in TAAD tissues. PCDH11X is a member of the protocadherin family involved in calcium-dependent cell–cell adhesion (40, 41). PCDH11X was notably downregulated in TAAD samples and exhibited positive correlation with protective immune cell subsets such as M2 macrophages. Its loss may impair intercellular adhesion among VSMCs or endothelial cells, facilitating medial layer disintegration. Intriguingly, the gene’s X-linked chromosomal location raises the possibility of sex-specific regulatory mechanisms, which merits further investigation given the known male predominance in TAAD incidence. Previous studies established that protocadherin family members regulate VSMC phenotypic switching via the Wnt/β-catenin pathway (42), suggesting PCDH11X may similarly contribute to vascular homeostasis maintenance. Intriguingly, the unique Xq21.31 chromosomal localization of PCDH11X could partially account for TAAD’s sex disparity features, providing new directions for sex-specific investigations (38, 39). These collective findings unveil novel molecular targets for deciphering TAAD pathogenesis. Importantly, our diagnostic model based on these three genes achieved excellent performance across multiple algorithms (AUC > 0.9), with consistent validation in independent datasets. While prior models have demonstrated diagnostic potential for TAAD using radiomic or proteomic features, few offer biological interpretability (43). The incorporation of SHAP values in our framework bridges the gap between predictive performance and mechanistic insight, enabling gene-level attribution of risk—a critical advancement for clinical translation.

Nevertheless, several limitations should be acknowledged. First, the transcriptomic data used in this study were derived from public repositories, introducing heterogeneity in sample collection and processing. Despite integrating datasets from multiple platforms with rigorous batch-effect correction and confirming its effectiveness via PCA, we recognize that residual technical biases cannot be fully ruled out. Sources such as platform-specific probe design or varying sequencing depths may introduce systematic errors that subtly affect gene expression metrics. Consequently, although steps were taken to minimize bias, prospective validation in independent cohorts is necessary. Second, although we demonstrated that SIX4 promotes VSMC proliferation and migration *in vitro*, *in vivo* studies using genetic models or patient-derived primary cells are necessary to validate its functional relevance in TAAD progression. This study substantiates the critical role of SIX4 in facilitating the proliferation and migration of VSMCs. However, the specific molecular pathways responsible for these effects, such as the potential involvement of AKT/HIF1α or CXCL12-CXCR4 signaling, as well as the detailed mechanisms by which SIX4 modulates the immune microenvironment, have yet to be comprehensively elucidated. The complexity of these mechanisms necessitates further dedicated investigation. Third, the clinical utility of our model—such as in blood-based diagnostics or prognostic stratification—requires further evaluation, particularly in comparison with established biomarkers like D-dimer or imaging parameters. While our study functionally validated SIX4 as a key regulator of VSMC behavior, future work should include functional assays for SCNN1B and PCDH11X to fully elucidate their roles in TAAD pathogenesis and further validate our multi-gene diagnostic model.

## 5 Conclusion

In this study, we have identified SIX4, SCNN1B, and PCDH11X as hub genes in TAAD, with SIX4 emerging as a key driver linking smooth muscle cell plasticity to immune dysregulation. Through multi-omics integration, single-cell analysis, and SHAP-enhanced modeling, we provide mechanistic insight and establish SIX4 as a promising biomarker and therapeutic target for early diagnosis and precision intervention in TAAD.

## Data Availability

The original contributions presented in the study are included in the article/**Supplementary Material**. Further inquiries can be directed to the corresponding authors.
